# cGAS-STING pathway as a potential trigger of immunosenescence and inflammaging

**DOI:** 10.3389/fimmu.2023.1132653

**Published:** 2023-02-28

**Authors:** Carine Raquel Richter Schmitz, Rafael Moura Maurmann, Fatima T. C. R. Guma, Moisés Evandro Bauer, Florencia Maria Barbé-Tuana

**Affiliations:** ^1^ Laboratório de Imunobiologia, Escola de Ciências da Saúde e da Vida, Pontifícia Universidade Católica do Rio Grande do Sul (PUCRS), Porto Alegre, Brazil; ^2^ Programa de Pós-Graduação em Ciência Biológicas - Bioquímica, Departamento de Bioquímica, Universidade Federal do Rio Grande do Sul, Porto Alegre, Brazil; ^3^ Programa de Pós-Graduação em Biologia Celular e Molecular, Escola de Ciências da Saúde e da Vida, Pontifícia Universidade Católica do Rio Grande do Sul (PUCRS), Porto Alegre, Brazil; ^4^ Instituto Nacional de Ciência e Tecnologia – Neuroimunomodulação (INCT-NIM), Conselho Nacional de Desenvolvimento Científico e Tecnológico (CNPq), Brasília, Brazil; ^5^ Programa de Pós-Graduação em Gerontologia Biomédica, Escola de Medicina, Pontifícia Universidade do Rio Grande do Sul (PUCRS), Porto Alegre, Brazil; ^6^ Programa de Pós-Graduação em Biologia Celular e Molecular da Escola de Ciências da Saúde da Vida, Pontifícia Universidade Católica do Rio Grande do Sul, Porto Alegre, Brazil; ^7^ Programa de Pós-Graduação em Pediatria e Saúde da Criança da Escola de Medicina, Pontifícia Universidade Católica do Rio Grande do Sul, Porto Alegre, Brazil

**Keywords:** aging, cGAS, immunosenescence, inflammaging, NF-κB, SASP, senescence

## Abstract

Aging is associated with an increased incidence of autoimmune diseases, despite the progressive decline of immune responses (immunosenescence). This apparent paradox can be explained by the age-related chronic low-grade systemic inflammation (inflammaging) and progressive dysregulation of innate signaling. During cellular aging, there is an accumulation of damaged DNA in the cell’s cytoplasm, which serves as ubiquitous danger-associated molecule, promptly recognized by DNA sensors. For instance, the free cytoplasmic DNA can be recognized, by DNA-sensing molecules like cGAS-STING (cyclic GMP-AMP synthase linked to a stimulator of interferon genes), triggering transcriptional factors involved in the secretion of pro-inflammatory mediators. However, the contribution of this pathway to the aging immune system remains largely unknown. Here, we highlight recent advances in understanding the biology of the cGAS-STING pathway, its influence on the senescence-associated secretory phenotype (SASP), and its modulation of the immune system during sterile inflammation. We propose that this important stress sensor of DNA damage is also a trigger of immunosenescence and inflammaging.

## Introduction

The aging immune system, *i.e.*, immunosenescence, has been associated with increased morbidity and mortality across the lifespan, of note in elderly populations. The progressive homeostatic imbalance of the immune system is accompanied by a higher incidence of autoimmune disorders, infections, and neoplasia (1–3). At the same time, a sterile and persistent low-grade inflammation (*i.e.*, inflammaging) contributes significantly to the physiological decline and development of age-related pathologies (2). Indeed, the interplay of immunosenescence and inflammaging significantly increases the risk for the development of diabetes, atherosclerosis, autoimmune diseases, cognitive impairment, neurodegenerative diseases, and cancer (4). However, the underlying mechanisms connecting immunosenescence and inflammaging remain unclear. One potential explanation could be the gradual accumulation of senescent cells in tissues alongside the impaired recognition and removal of those by the aging immune system.

Senescent cells are powerful stimulators of immune cells, triggering local and systemic inflammation by actively releasing pro-inflammatory mediators and stress signals (2, 5). The recognition and subsequent clearance of these cells by the immune system are crucial to restrain inflammation and promote tissue regeneration (6). During cellular aging, there is an accumulation of damaged DNA in the cell’s cytoplasm which can be promptly recognized by DNA sensors. In this context, the stress sensor pathway mediated by the cyclic guanosine monophosphate-adenosine monophosphate (cGAMP) synthase (cGAS) and the adaptor stimulator of interferon genes (STING) emerges as an important link between senescence and immune response (7, 8). The cGAS-STING pathway has been reported to be critically involved in autoimmune and chronic inflammatory disorders, orchestrating a connection between immune responses against DNA damage-derived stimuli (8, 9). In this review, we summarize the current knowledge on the biology of the cGAS-STING pathway during aging and we discuss its involvement in the context (regulation) of immunosenescence and inflammaging.

## Cellular senescence as a source of alarmins

Cellular senescence is considered an underlying cause of physiological aging and it has been implicated in all age-related diseases (5). The senescent phenotype is characterized by a stable cell cycle arrest accompanied, extensive macromolecular damage, an altered active metabolic state with a distinct secretory profile (10, 11). While it represents a physiological stress response attempted to restrain cellular damage and mediate tissue repair (6), the progressive accumulation of senescent cells has a detrimental effect on tissue physiology during aging (12, 13).

Senescent cells typically engage a DNA damage response (DDR), defined by increased recruitment of DNA damage signaling histones (*e.g.*, phosphorylated H2AX; γH2AX) and the DNA damage repair that directs protein complexes to damaged sites of DNA (11, 14). The accumulation of these *foci* observed in aged tissues correlates with persistent chromatin alterations (14), which further relates to the disruptive remodeling of the nuclear lamina, *i.e.*, decreased expression of Lamin B1 (15, 16). In addition, a progressive redistribution and loss of heterochromatin (16), as well as an increased genomic instability and altered global gene expression, are observed (17, 18).

Defective DDR driving genomic and nuclear instability accounts for the accumulation of micronuclei and cytoplasmic chromatin fragments (CCF) or cytosolic DNA fragments (10, 19–22). These dsDNA fragments constitute potent pro-inflammatory stimuli, driving the senescence-associated secretory phenotype (SASP) (10, 23), a hallmark of senescent cells (5). SASP contemplates a dynamic and varied set of pro-inflammatory cytokines and chemokines (*e.g.*, IL-1α, IL-6, IL-8, and TNF-α), growth and angiogenic factors (*e.g.*, epithelial growth factor [EGF], vascular epithelial growth factor [VEGF], and insulin growth factor binding protein [IGFBP] 1 and 2), and matrix metalloproteinases (MMPs | *e.g.*, MMP1, MMP3, MMP12 and tissue inhibitor of metalloproteinase 1 [TIMP-1]) ultimately orchestrated by the nuclear factor ĸB (NF-ĸB), CCAAT/enhancer-binding protein β (C/EBPβ), bromodomain-containing protein 4 (BRD4), lysine methyltransferase MLL1 and G9A (24). While it is true that SASP is involved in the recruitment and activation of immune cells for senescence clearance (5, 6), it also exerts a direct effect on wound healing and tissue regeneration (25, 26). However, persistent SASP secretion leads to altered tissue structure and function (10, 27), dysregulated tissue metabolism (28), and accelerated aging process (12). SASP is further aggravated by the downregulation of deoxyribonucleases (DNases) (29) which potentiates cytoplasmic accumulation of double-strand DNA (dsDNA) and SASP secretion (29, 30). Both secreted SASP factors and cytoplasmic dsDNA act during normal or accelerated aging as potent triggers of sterile inflammation (28, 31). Indeed, the crosstalk of damage-associated molecular pattern (DAMP) sensors and persistent macromolecular damage may constitute an important link between inflammaging and senescence (19, 32, 33).

SASP modulation of tissue microenvironment is crucially connected with impaired immune function and inflammaging (2, 34). A notorious aspect of SASP is its potential for reinforcing and spreading senescence through autocrine and paracrine pathways (5), affecting local cells and, in particular, immune cells (*e.g.* resident macrophages, Mφ) (35, 36). Indeed, increased levels of pro-inflammatory mediators derived from SASP correlate to Mφ function decline similarly to observed in aging (37, 38). Moreover, selective elimination of senescent cells improves immune functions in peripheral compartments (39), corroborating the potential systemic deleterious effect of exacerbated SASP secretion (2). In parallel, senescent immune cells, especially Mφ, present ineffective clearance capacity promoting prolonged local inflammation and eventual systemic extrapolation, therefore contributing to inflammaging (2, 28).

## The cGAS-STING pathway: A regulator of inflammation

The innate immune cells respond to pathogens and damaged tissues *via* recognition of pathogen-associated molecular patterns (PAMPs) and DAMPs, respectively (40). Among danger molecules, self-DNA is a potent alarming molecule. Both endogenous molecules (ROS) or exogenous damaging agents may induce single or double strand DNA breaks (ssDNA or dsDNA) that escape to the cytoplasm. The cytoplasmic DNA generates an alarm signal that, if unrepaired, can induce inflammation (41). Sterile inflammation triggered by the recognition of self-content has been greatly expanded with the discovery of new sensors and recently extensively reviewed (31).

In this context, the cGAS-STING pathway emerges as a central node modulating the expression and activity of DAMP sensors and eliciting optimal immune function (42, 43). This pathway integrates immune sensing of DNA damage and cfDNA derived from senescent cells (10, 44). The cGAS protein is the major cytoplasmic dsDNA recognition sensor, recognizing the cell’s DNA when damaged and outside its subcellular compartments, such as the nucleus and mitochondria (45). Disrupted cGAS-STING signaling accounts for defective DAMP sensing and impaired immune activation (46, 47), which may contribute to cumulative senescence and SASP-derived inflammation (48, 49). This connection, however, has not been directly addressed, representing an open avenue to be explored and a major focus of interest of this review. The cGAS-STING are expressed by a variety of immune and non-hematopoietic cells, including Mφ, dendritic cells, natural killer (NK) cells, T and B cells, endothelial cells, epithelial cells, and neurons (50).

**Figure 1:**
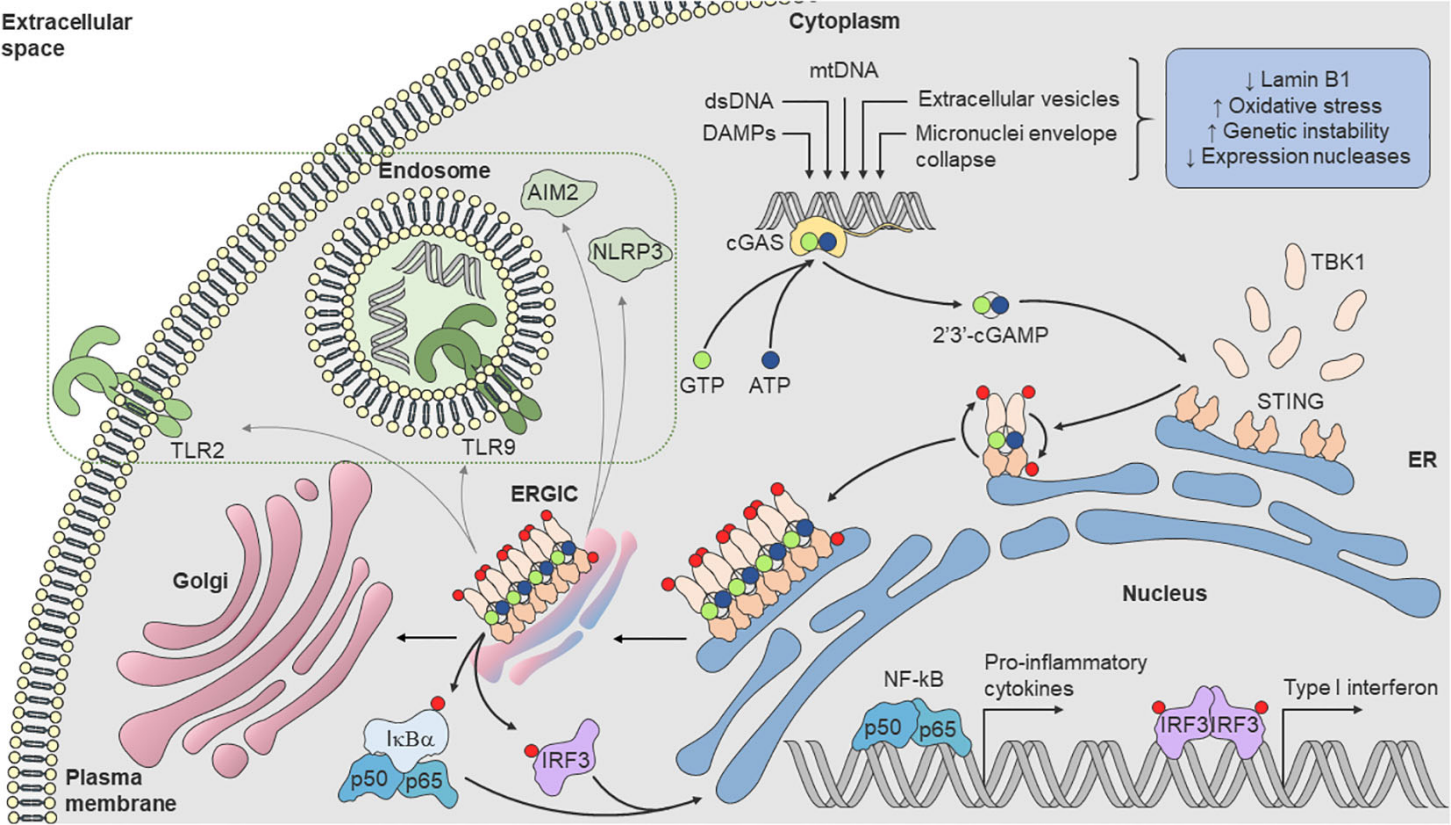
cGAS-STING pathway. Loss of Lamin B1, increased oxidative stress, genomic instability, mitochondrial dysfunction, as well as reduced expression of nucleases, lead to dsDNA break and accumulation in cytosol. Cytoplasmic cGAS recognizes dsDNA and synthetizes the endogenous secondary messenger 2’3’-cGAMP, which in turn, activates the ER transmembrane protein STING. cGAMP binds to STING, oligomerizes and becomes active with translocation to ERGIC. During translocation, STING recruits TBK1 [(D or E)xPxPLR(S or T)D] motif (in which “x” denotes any amino acid) and induces its phosphorylation, leading to the recruitment of IRF3. TBK1 phosphorylates IRF3, leading to dimerization and translocation into the nucleus, where it induces transcription of genes encoding various cytokines, chemokines, and interferons. Activated TBK1 also phosphorylates IκBα, the inhibitor subunit of the IKK complex, promoting the release of the heterodimer p50/p65 (NF-κB), which translocates with IRF3 into the nucleus, providing a synergistic pro-inflammatory response. STING can also undergo numerous conformational changes that can also trigger the activation and increased expression of AIM2 and NLRP3 inflammasomes and TLR2 expression and TLR9 pathway response (dashed rectangle). AIM2, absent in melanoma 2; cGAS, cyclic GMP–AMP synthase; 2’3’-cGAMP, cyclic guanosine monophosphate-adenosine monophosphate; ER, endoplasmic reticulum; ERGIC, endoplasmic-reticulum–Golgi intermediate compartment; IKK, inhibitor κB kinase; IκBα, NF-κB inhibitor α subunit; IRF3, interferon regulatory factor-3; NF-κB, nuclear factor κB; NLRP3, nucleotide-binding oligomerization domain-like receptor protein 3; STING, stimulator of interferon genes; TBK1, TANK-binding kinase-1; TLR, Toll-like receptor.

STING was first described in 2008 (51). It is a transmembrane homodimer protein localized in the endoplasmic reticulum (ER) previously reported to participate in innate immune signaling against viral pathogens. STING is activated upon cGAMP binding, oligomerization, and translocation to ERGIC. STING inducing the activation of transcription factors IRF3 and NF-κB and drives the secretion of type I interferons (IFN-α and IFN-β) and inflammatory cytokines (51, 52). However, its connection to cGAS was only established in 2013, identified as a cytosolic DNA sensor responsible for synthesizing cGAMP, the second messenger that activates STING (53–55). Since then, the cGAS-STING pathway has been considered a major pathway for cellular stress that senses self and non-self DNA and connects DNA damage to inflammation (**Figure 1**), modulating pro-inflammatory cytokines and type I interferons (IFN-I) production (7, 8). In this sense, the cGAS-STING pathway is implicated in multiple age-related diseases, including autoimmune diseases (9) and neoplasias (10, 32).

cGAS-STING activation may act both in favor and against senescence spreading. Functional cGAS-STING is required for optimal response in immune cells (42, 43). For instance, STING modulates the expression of NKG2D, an activating cell surface receptor, present on NK and T cells and associated with immune surveillance (56). In parallel, STING activity in senescent and cancer cells do also mediate the expression of NKG2D ligands, favoring immune recognition (57, 58). At the same time, STING activation is implicated in the senescence associated secretory phenotype (SASP) production and its inhibition shows a significant reduction in the expression of pro-inflammatory mediators in senescent cells (33). Defects identified in cGAS or STING interfere with the expression of distinct components of SASP and lead to deficient activation of immune cells due to loss of responsiveness to cGAMP (46). Indeed, the significance of cGAS has been demonstrated in mouse embryonic fibroblasts deficient in cGAS (cGAS^−^/^−^). In this context, knock-out cells exhibited reduced signs of senescence (59).

On the other hand, STING downregulation is correlated with cancer progression (60). Inhibition of the cGAS-STING pathway may facilitate the escape of cancer cells from immunosurveillance (10, 43). For example, the proportions of CD3^+^, CD4^+^, and CD8^+^ T cells producing IFN-γ were significantly lower in double-deficient cGAS-STING tumors (61). In contrast, STING activation triggered the migration of Mφ to tumors and induced CD4^+^ and CD8^+^ T-cell infiltration by increased chemokine secretion (62–64). STING agonists may also lead cancer cells to apoptotic death (65) and initiate subsequent clearance by phagocytic cells, thus promoting increased immune cell recruitment and decreased susceptibility for developing new tumours (29, 66). Ultimately, cGAS or STING expression were found reduced in many cancers cell lines (67), a possible mechanism to circumvent the immune system.

The STING pathway has been described as essential for CD4^+^ and CD8^+^ lymphocytic infiltration into tumors, governed by IFN-α and IFN-β and other chemokines, like CCL5 and CXCL10 (63, 68–72). STING agonists might facilitate the activation of dendritic cells (DCs) and priming and infiltration of cytotoxic T cells through CXCR3 ligands (68, 73). On the other hand, STING knockdown inhibited the nuclear import of the Nuclear Factor NF-Kappa-B p65 subunit and IRF3 and restricted the secretion of the CXCL10 and IL-6 in an odontoblast−like cell line (74).

STING activation may yet be associated with exacerbated pro-inflammatory responses of Mφ in chronic inflammatory diseases, such as non-alcoholic fatty liver disease (NAFLD) and hepatic fibrosis (66, 75). STING disruption in NAFLD promotes decreased Mφ pro-inflammatory activation and exhibits marginal effects on Mφ alternative (M2) activation (66). In addition, an enrichment analysis showed that activation of the cGAS-STING pathway increased proteins involved in cell cycle control of chromosomal replication, mitochondrial dysfunction, sirtuin signaling, protein ubiquitination, antigen processing, and Fc receptor-mediated phagocytosis in Mφ (76). In a silica-induced lung inflammation model, an important role to self-dsDNA released upon silica-induced injury was demonstrated activating the pro-inflammatory response by IFN-I and CXCL10 in a STING-dependent manner (55).

## Inflammaging: A fuel for cGAS

Aging is associated with a chronic low-grade sterile inflammation termed inflammaging (2, 77). While its origins remain unclear, inflammaging is typically characterized by high serum levels of pro-inflammatory cytokines (*e.g.*, tumour necrosis factor α [TNF-α], interleukin 6 [IL-6], and IL-1), chemokines (*e.g.*, monocyte chemoattractant protein [MCP] and IL-8), acute phase proteins (*e.g.*, C-reactive protein [CRP]), and soluble cytokine receptors (*e.g.*, soluble tumour necrosis factor receptor [sTNF-R] I and II) (2, 78). Inflammaging has been directly associated with the systemic physiological decline observed in aging, representing an increased risk of morbidity and mortality *per se* (2, 79). Despite its multiple triggers, the inflammaging is frequently understood as a result of multiple age-related changes linked to the progressive accumulation of cellular damage (80, 81). Chronic sterile inflammation is triggered by DAMPs (endogenous molecules including proteins and DNA) which can be altered, damaged, or misplaced inside the cell (45). Senescent cells have increased levels of damaged mtDNA or nDNA, located outside their cellular compartments, triggering inflammaging and thus contributing to SASP and increased production of pro-inflammatory cytokines.

Inflammaging and immunosenescence are not the same, but classically interconnected and linked by a vicious cycle of overstimulation and functional impairment (81). For instance, chronic stimulation by cytokines, chemokines, and cell debris derived from dyeing or damaged aged tissues correlates with T cell exhaustion, cellular senescence, and a dysfunctional pro-inflammatory phenotype (82, 83). Increased proportion of exhausted and senescent T cells, in its turn, is related to augmented susceptibility to infections, neoplasia, and chronic inflammatory disorders, which further contributes to systemic inflammation (2, 84).

Inflammaging has been implicated in a plethora of age-related conditions and disorders like obesity (85–87), type II diabetes (85, 88), atherosclerosis (89), autoimmune diseases, cognitive impairment, neurodegenerative diseases (90–93), and cancer (94). In addition, the chronic inflammation observed in these disorders equally contributes to a systemic physiological decline similarly observed in the elderly, reinforcing the deleterious effect of this phenomenon *per se* (91, 95). A causal link between the inflammatory status and pathogenesis is also established in chronic respiratory diseases (93), fatty liver disease (66, 92) and osteoarthritis (93).

Considering the multiple elements contributing to inflammaging-like conditions, a critical point is to understand why they are reinforced and exacerbated in aging and age-related diseases. In this sense, cellular senescence emerges as a central mediator, integrating age-related cumulative cellular damage (e.g., DNA damage) and chronic inflammation (79, 80). In fact, the aberrant activation of cGAS-STING is a feature of senescent cells and critical for the induction of senescence. The depletion of cGAS in mouse embryonic fibroblasts (MEF) has been shown to inhibit the secretion of cytokines of the SASP phenotype (IL-1β, IL-6 and metalloprotease-12) in a similar manner to the action of two anticancer agents for DNA damage, etoposide or irradiation (59). Indeed, the elimination of senescent cells has been shown to improve several bodily tissue functions *via* reducing pro-inflammatory cytokines and chemokines (27), in parallel with a concomitant improvement of immune cell functions (39). However, the complex mechanisms linking senescence, inflammaging, and immunosenescence are still unclear.

## Crosstalk between cGAS-STING and DAMP sensors

Receptors to endogenous host-derived molecules (DAMPs) are expressed by innate immune cells and are highly activated in sterile inflammation (31). Aging is associated with DAMP receptor dysregulation, which may contribute to increased morbidity and mortality (96). In particular, many cytosolic and endosomal receptors recognize self-DNA accumulation beyond cGAS, such as absent in melanoma 2 (AIM2), NOD-like receptor P3 (NLRP3) and TLR9, or extracellular cell free DNA (cfDNA), and TLR4 (30, 31, 97–99). These receptors not only share intracellular pathways but are also interconnected with STING activity (31, 43, 100).

Experiments of gain/loss of function in the context of oncogene-induced senescence (OIS) have shown that TLR2 transcription is repressed on silenced STING cells while its expression and activation seem to be downstream of the cGAS-STING node (43). In human monocytes, TLR2 activation upregulates NLRP3 expression (101). Moreover, TLR9 and STING stimulation induce IFN-γ production synergistically (102). Stimulation of cGAS-STING also increases the suppressor of cytokine signalling 1 (SOCS1) and SOCS3 (103), involved in targeting IRF7 degradation, an activator of TLR9 (75). In addition, decreased capacity to secrete IFN-I results in impaired phosphorylation of the IRF7 transcription factor (104). cGAMP is also described as an AIM2 (43) and NLRP3 inflammasome activator (8), being STING required for optimal cGAMP-induced inflammasome function (100, 105, 106). Moreover, it has been shown that STING colocalizes with AIM2 and is required for IL-1β secretion and caspase-1 activation (107). cGAMP may also activate inflammasomes *via* an AIM2-NLRP3-ASC-dependent pathway (100). On the other hand, inflammasome activation has been shown to negatively regulate cGAS activity by caspase cleavage of cGAS (108). Hence, AIM2 inflammasome can attenuate cGAS–STING-mediated IFN-I production in viral infections while Mφ DKO (*Asc^−/−^* and *Caspase 1^−/−^*) increase IFN production upon DNA virus infection, suggesting a cross-regulation between these pathways. STING has been implicated in the antiviral response. For example, the deletion of Atm (*Atm*-/-), a kinase that plays a central role in the DNA repair pathway, induces the accumulation of self-damaged DNA in the cytoplasm and increases the expression of type I IFNs. This signaling enhances the anti-microbial response in neighboring cells through STING by priming the antiviral innate immune response upon microbial challenge. In this model, the accumulation of DNA damage increases the expression of IFN-I, a Tlr4, Tlr2, and Rig-I downstream Irf7, and STING, as observed in patients with the neurodegenerative disease AT (109). Taken together, these results demonstrated that the cGAS-STING pathway could be considered an important central node of communication between DAMP sensors and its optimal activity. On the other hand, failure to activate cGAS-STING signaling might downregulate directly or indirectly this same DAMP receptors’ activity and expression.

## Immunosenescence: Focus on T-cells and macrophages

Immunosenescence is characterized by an age-dependent remodeling of the immune system. It involves structural alterations of lymphoid organs as well as phenotypical and functional changes in peripheral immune cells (110). This process is marked by the progressive loss of adaptive immune function and the relative conservation and overcompensation of innate immunity (1, 81), which is associated with a state of increased autoimmunity, immunodeficiency and sterile inflammation (2, 3, 81).

The hematological decline of adaptive immunity initiates after adolescence when there is a progressive thymus involution and consequently reduced export of naïve T cells (CD27^+^CD28 ^+^CD45RA^+^) from the thymus (1). The shrinkage of the naïve T-cell compartment as well as memory inflation with increased expansion of senescent T cells (CD27^-^CD28^-^CD57^+^CD45RA^+^) is associated with reduced immune responses to vaccination and impaired immunity against infections in older adults (81, 111, 112). Some immunosenescent changes are particularly well observed later in life. Indeed, after the sixth decade of life, there is a robust contraction of the T-cell diversity and clonal expansion, drastically limiting the recognition of new antigens by T cells (113).

In contrast, the innate immunity remodeling during aging is characterized by an increased basal level of stimulation associated by disrupted specific functions (*e.g.*, phagocytosis) (34). In this context, Mφ emerge as critical players due to their central role in maintaining tissue homeostasis (2, 37). Aged Mφ present a dysregulated pro-inflammatory response to local damage alongside an ineffective resolution potential, thus favouring the exacerbation of tissue inflammation (37, 77). Failure in Mφ function can result in a gradual increase of low-grade physiological inflammation and an aberrant adaptive immune cell activation contributing to morbidity and mortality in advanced age (114). Furthermore, resident Mφ accumulate cellular damage (77, 115) concomitant with diminished expression of Toll-like receptors (TLRs) and major histocompatibility complex (MHC II), which provides an ineffective recognition of invading pathogens and commensal flora (114). Recent data have demonstrated that long-term selective elimination of Mφ in aging mice decreased pro-inflammatory cytokine concentrations while systemic immune stimulation (high-dose anti-CD40/IL-2 or IL-2/IL-12) led to early death (38, 77). In line with these results, it was observed that aged-foamy Mφ accumulate in the sub-endothelial space, driving the atherosclerotic process through increased expression of inflammatory cytokines, chemokines, and metalloproteinases (116). The involvement of Mφ in developing age-related diseases (17, 37, 117) and maintaining inflammation has been widely explored and has been termed “macroph-aging” (37).

The age-dependent decline of Mφ function correlates with alterations in the aged tissue microenvironment, delineated by increased levels of pro-inflammatory mediators and stress signals (38). These persistent immune triggers may drive metabolic and functional alterations in resident Mφ irrespective of their ontological origin (38, 118). For instance, while transplanted peritoneal Mφ from old to young mice show similar phagocytic function as young-derived Mφ, transplanted peritoneal Mφ from young to old animals have impaired phagocytosis acquiring features of aged Mφ (119). In addition, embryonic-derived resident Mφ are gradually replaced by bone marrow-derived monocytes, which in turn have a potent pro-inflammatory signature in the elderly, further enhancing tissue inflammation (36, 120).

## Connecting the cGAS-STING pathway to immunosenescence and inflammaging

**Figure 2:**
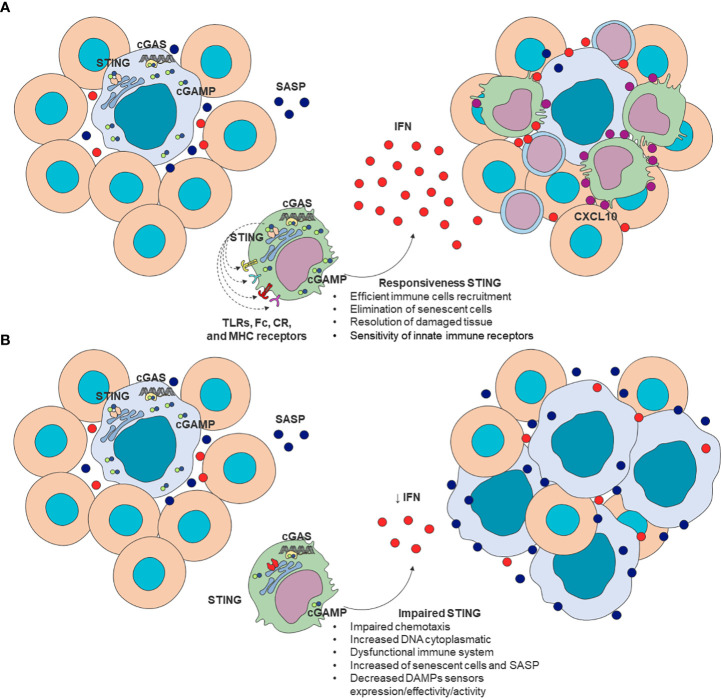
A proposed model of STING as a modulator of immunosenescence. **(A)** Effective STING response is required for efficient immune cell recruitment, elimination of senescent cells, and damaged tissue repair. **(B)** Impaired STING signaling can be associated with increased cytoplasmatic DNA, impaired chemotaxis, decreased DNA sensors’ expression and effectivity, increased frequency of senescent cells and SASP mediators leading to impaired clearance of senescent cells. These alterations are consistent with changes present in aged-immune cells. SASP (blue); T cells chemokines (purple); IFN (red). cGAS, cyclic GMP–AMP synthase; 2’3’-cGAMP, cyclic guanosine monophosphate-adenosine monophosphate; SASP, senescence-associated secretory phenotype; STING, stimulator of interferon genes.

Despite many research efforts, some crucial questions remain to be answered in gerontology: what are the triggers of immunosenescence? How does immunosenescence contribute to inflammaging and vice versa? Are the phenotypical and functional age-related changes in immune cells intrinsic to immunosenescence or derived from aging tissues? In this context, the cumulative macromolecular damage derived from senescent cells, especially in DNA damage, and its implication on inflammation and immune activation seems to be a central element (48, 121). In particular, its connection to the cytosolic DNA sensing system mediated by cGAS-STING seems to provide a direct link with immunosenescence (10). While functional STING is required for optimal immune function (42, 43) and its disruption is associated with reduced sensitivity of innate immune receptors (47), which could be related to impaired senescence clearance (49). Considering that these events have common characteristics associated with immunosenescence (49, 114), defective cGAS-STING signaling may represent a central node in the age-related transition of immune function decline and accumulation of senescent cells (**Figure 2**).

Following these hypotheses, a recent link between immunosenescence and senescent somatic cell (SSC) accumulation has been established (122, 123). For instance, reduced age-related chemotaxis and phagocytosis in Mφ and neutrophils seem to correlate with impaired elimination of SSCs. Besides, cytotoxic T cells may also mediate SSC clearance directly or by releasing inflammatory cytokines such as IFN-γ, functions equally disrupted (123). The dysregulation of these mechanisms may ultimately result in increased SASP production, contributing to inflammaging (81, 122, 123).

Although direct connections between the cGAS-STING node and immunosenescence remain barely explored, a plethora of indirect evidence may sustain this idea. STING knockout and inhibition studies, in particular, demonstrate impaired DAMP recognition due to disruptive function of their receptors in immune cells (43, 46, 47, 108). In this sense, aged Mφ presents reduced NLRP3 inflammasome activation (124) as well as AIM2 expression and ligand-dependent activity in whole blood cells (104). In addition, TLR functions were found impaired in multiple immune cells in the elderly (96, 104). On the other hand, TLR, AIM2, and NLRP3 expression were found to increase in non-immune tissues (124–127), which seems in accordance with STING activation in non-immune senescent cells (33, 46). Taken together, these data suggest altered cGAS-STING signaling may be a plausible player involved in the age-related changes in DAMP receptors.

Moreover, it is noteworthy to mention that aged immune cells have a limited capacity to secrete IFN-I (128, 129), a cGAS-STING-related function (52). Elderly-plasmacytoid dendritic cells (pDCs), for instance, secrete lower levels of IFN-I after stimulation because of age-associated impaired IRF7 phosphorylation. Besides, pDCs present reduced capacity to present antigens to CD4+ and CD8+ T lymphocytes while retaining a certain production of pro-inflammatory cytokines that activate CD8+ T cells (130). Despite IFN production may be modulated by cGAS-STING and TLR, the former accounts for the majority of IFN secretion upon stimulation (126, 131), while its knockout significantly compromises IFN signaling (132). Interestingly, increased expression of IL-6, a marker of inflammaging was shown to promote STING degradation following dsDNA stimulation (133), which may reinforce the link between inflammaging and STING impaired function.

**Figure 3:**
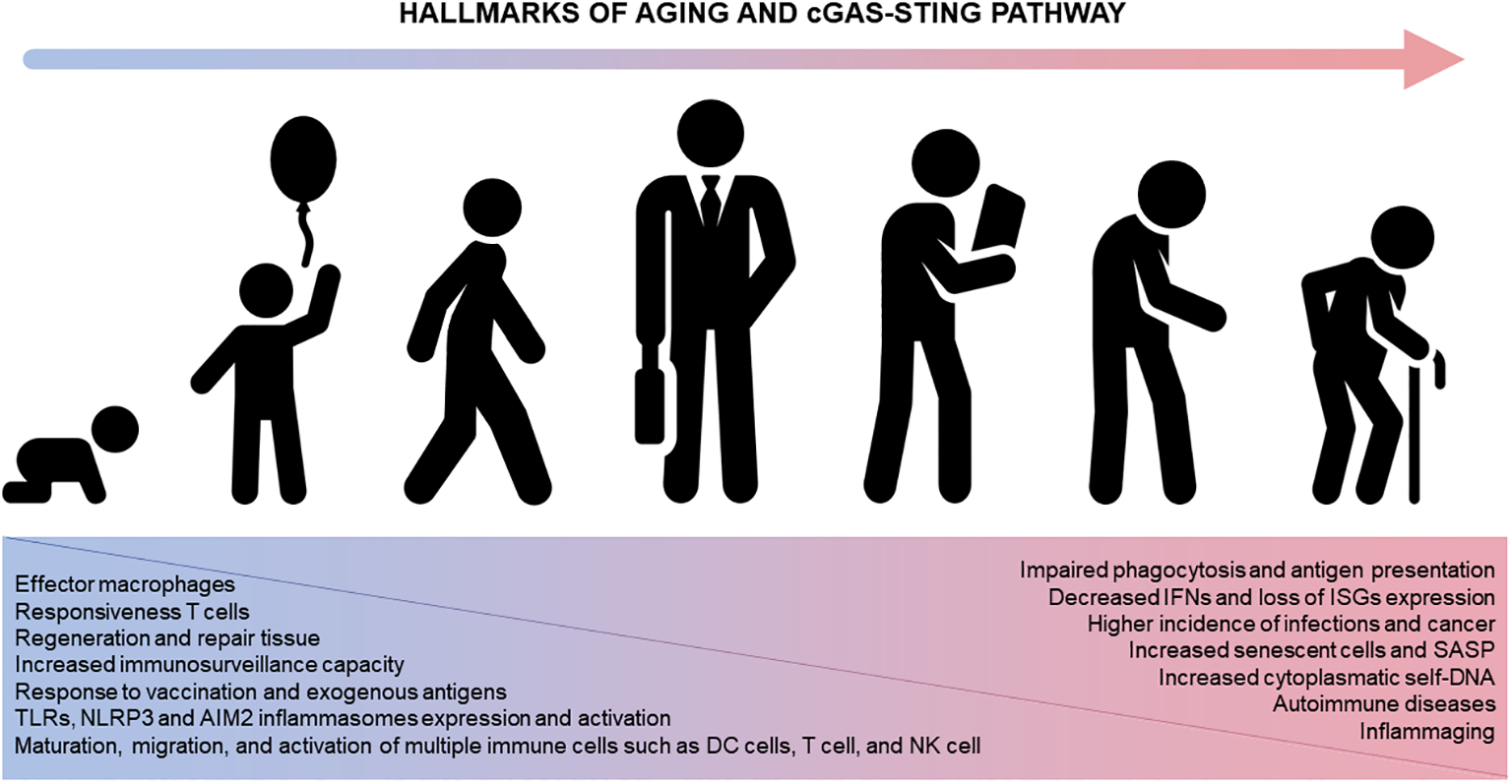
The cGAS-STING pathway and aging. STING participates in numerous immune system functions (left, blue). Physiological alterations in the immune system across lifespan ultimately compromise the immune system homeostasis impacting different diseases and disorders (right, pink). AIM2, absent in melanoma 2; DC, dendritic cells; ISG, IFN-stimulating genes; IFN, interferon; NK, natural killer cells; NLRP3, nucleotide-binding oligomerization domain-like receptor protein 3; TLR, Toll-like receptors.

Immunosenescence also correlates with a higher incidence of autoimmune disorders (134) and neoplasia (135) as well as poor vaccine response (1–3). In this regard, STING knockout animals present attenuated inflammation in autoimmune diseases (136, 137), characterizing a potential therapeutic target with small-molecules STING antagonists ready for clinical trials (138). In contrast, the lack of STING signaling has been associated with increased cancer during aging and specific mutations compromising STING expression can prevent DNA damaged cells from producing pro-inflammatory cytokines and alert the immune system (60, 139). Pharmacological activation of the STING pathway has been shown important in T-cell mediated tumor regression (140). Furthermore, STING agonists (*e.g.*, MK-1454 and ABZI) have been tested successfully in advanced/metastatic solid tumors and lymphomas (141). In parallel, some isoforms of cGAMP were identified as effective vaccine adjuvants, being useful for developing novel immunotherapies with protective antiviral CD8^+^ T cell responses (142). In summary, we propose cGAS-STING as important intracellular pathway crucially involved with the hallmarks of aging as well as development of immunosenescence and inflammaging (**Figure 3**).

Nonetheless, convincing evidence to corroborate the causal link between cGAS-STING and immunosenescence as well as to elucidate the consequences of its dysregulation is still required. This is particularly of note in autoimmune and chronic inflammatory diseases where STING activation further aggravates inflammation (9, 66). Furthermore, STING mediates calcium homeostasis disruption in T cells triggering cell death (143), while STING ligands (*e.g.*, cGAMP and DMXAA) inhibited the proliferation of naïve CD4+ T cells by mTORC1 and IFN-I signaling pathways (144), unexpected outcomes associated with this node. These discrepant results could be explained by the presence of numerous molecules involved in cGAS-STING modulation (75, 103, 145). For instance, TOLLIP was found to positively regulate STING-mediated immune response while Tollip ^–/–^ mice presented reduced STING expression with a more pronounced effect in nonhematopoietic tissues (103). Besides, gamma-interferon-inducible protein (IFI16) was also found to be required for cGAMP-induced activation, phosphorylation, and translocation of STING in human keratinocytes (146, 147) and Mφ (147, 148). On the other hand, p38-MAPK-mediated phosphorylation of USP21, a deubiquitinating protein, induces STING deubiquitination blocking STING-TBK1-IRF3 complex formation and inactivating IFN-I signaling (149, 150). The intracellular concentration of cGAMP may yet determine STING translocation or condensation (151), being high levels of this second messenger associated with downregulation of innate immunity and lowered IFN-I production (152). These interactions may be thus dependent on the cellular responses to metabolic processes and the cellular type and activation state (103).

## Concluding remarks

Aging is accompanied by the accumulation of non-repaired cellular damage, stem cell exhaustion, and phenotypic and metabolic changes – all of which are considered drivers of chronic inflammatory diseases, immunosenescence, and cancer. As the immune system is crucially involved in tracking and eliminating senescent cells (81, 123), dysfunctional immune mechanisms of clearance liaise with persistent and cumulative cellular damage, thus favoring a low-grade inflammation (inflammaging).

In this context, the cGAS-STING emerges as a new pathway that connects cellular damage and inflammation. Because cGAS-STING is a signal amplifier to produce IFNs in sterile inflammation, we propose that the low immunogenicity of immune cells in responding to cellular damage presented in aging could be connected to the malfunctioning of cGAS-STING signaling. New findings suggest that its relationship with other DAMPs´ sensors might explain decreased expression and activity of innate sensors during aging. As many molecular interactions are yet unclear, the modulation of STING and its impairment remain uncertain. However, the crystal resolution of the cGAS-dsDNA structures report the design of a new class of inhibitors that compete with ATP/GTP and bind to the active site (153), or compete with dsDNA (154) suggesting potential venues for intervention. Therefore, new studies are essential to understand the activation of the cGAS-STING pathway to shed light on the understanding and the interplay between immunosenescence and inflammaging as well as develop new therapies.

## Author contributions

CR and FMBT designed the review. CR, RM, MB and FBT were involved in writing the manuscript. All authors revised the manuscript and approved the final submitted version.
